# Relationship among porcine lncRNA TCONS_00010987, miR-323, and leptin receptor based on dual luciferase reporter gene assays and expression patterns

**DOI:** 10.5713/ajas.19.0065

**Published:** 2019-07-01

**Authors:** Yueyun Ding, Li Qian, Li Wang, Chaodong Wu, DengTao Li, Xiaodong Zhang, Zongjun Yin, Yuanlang Wang, Wei Zhang, Xudong Wu, Jian Ding, Min Yang, Liang Zhang, Jinnan Shang, Chonglong Wang, Yafei Gao

**Affiliations:** 1 Anhui Provincial Laboratory of Local Animal Genetic Resource Conservation and Bio-Breeding, College of Animal Science and Technology, Anhui Agricultural University, Hefei, Anhui 230036, China; 2 Key Laboratory of Pig Molecular Quantitative Genetics of Anhui Academy of Agricultural Sciences, Anhui Provincial Key Laboratory of Livestock and Poultry Product Safety Engineering, Institute of Animal Husbandry and Veterinary Medicine, Anhui Academy of Agricultural Sciences, Hefei, Anhui 230031, China; 3 Anhui Haoxiang Agriculture and Animal Husbandry Co. LTD, Bozhou, Anhui 236700, China

**Keywords:** Pig, *LEPR*, LncRNA TCONS_00010987, MiR-323, Dual Luciferase Reporter, Expression Patterns

## Abstract

**Objective:**

Considering the physiological and clinical importance of leptin receptor (*LEPR*) in regulating obesity and the fact that porcine *LEPR* expression is not known to be controlled by lncRNAs and miRNAs, we aim to characterize this gene as a potential target of *SSC*-miR-323 and the lncRNA TCONS_00010987.

**Methods:**

Bioinformatics analyses revealed that lncRNA TCONS_00010987 and *LEPR* have *SSC*-miR-323-binding sites and that *LEPR* might be a target of lncRNA TCONS_00010987 based on *cis* prediction. Wild-type and mutant TCONS_00010987-target sequence fragments and wild-type and mutant *LEPR* 3′-UTR fragments were generated and cloned into pmiR-RB-REPORT^TM^-Control vectors to construct respective recombinant plasmids. HEK293T cells were co-transfected with the *SSC*-miR-323 mimics or a negative control with constructs harboring the corresponding binding sites and relative luciferase activities were determined. Tissue expression patterns of lncRNA TCONS_00010987, *SSC*-miR-323, and *LEPR* in Anqing six-end-white (AQ, the obese breed) and Large White (LW, the lean breed) pigs were detected by real-time quantitative polymerase chain reaction; backfat expression of LEPR protein was detected by western blotting.

**Results:**

Target gene fragments were successfully cloned, and the four recombinant vectors were constructed. Compared to the negative control, *SSC*-miR-323 mimics significantly inhibited luciferase activity from the wild-type TCONS_00010987-target sequence and wild-type *LEPR*-3′-UTR (p<0.01 for both) but not from the mutant TCONS_00010987-target sequence and mutant *LEPR*-3′-UTR (p>0.05 for both). Backfat expression levels of TCONS_ 00010987 and *LEPR* in AQ pigs were significantly higher than those in LW pigs (p<0.01), whereas levels of *SSC*-miR-323 in AQ pigs were significantly lower than those in LW pigs (p<0.05). LEPR protein levels in the backfat tissues of AQ pigs were markedly higher than those in LW pigs (p<0.01).

**Conclusion:**

*LEPR* is a potential target of *SSC*-miR-323, and TCONS_00010987 might act as a sponge for *SSC*-miR-323 to regulate *LEPR* expression.

## INTRODUCTION

Obesity is a major source of human morbidity and mortality and is becoming more common worldwide [1]. Excess fat accumulation is a contributing factor to severe human diseases such as type II diabetes, cancers, and cardiovascular disease [2]. Therefore, it is important to understand the detailed mechanisms controlling adipogenesis and energy homeostasis. Currently, animal models for obesity research mainly are pigs, mice, and rats [3–5]. The pig is emerging rapidly as a biomedical model of energy metabolism and obesity in humans because it is postnatally devoid of brown fat and because of similar metabolic features, cardiovascular systems, and proportional organ sizes [6].

The products of the leptin receptor (*LEPR*) gene belong to the class I cytokine receptor family and were first reported in mice [7]. Poor expression of *LEPR* in certain tissues was suggested to lead to leptin resistance, which is commonly associated with obesity [8]. Ban et al [9] concluded that the knockdown of *LEPR* affects the mRNA expression of its downstream genes, suggesting that chicken *LEPR* plays a certain role in regulating the complicated gene network of preadipocytes. In pigs, a non-synonymous exonic polymorphism in the *LEPR* gene has been reported to be strongly associated with fat content in Iberian×Landrace and in Duroc×Landrace/Large White crossbreds [10]. The porcine *LEPR* gene is located between the 3rd subband and the 5th subband of the long arm 3 region of chromosome 6 and is located at the quantitative trait locus that is related to backfat thickness [11]. Compared to that of pigs, the nucleic acid homology of human and mouse *LEPR* sequences are 89.2% and 80.3%, and the amino acid sequence alignment is 86.5% and 76.6%, respectively [12]. *LEPR* has thus been considered a genetic marker associated with body composition, growth rate, and obesity in some pig breeds [13].

LncRNAs are greater than 200 nt in length and have been implicated in diverse biological processes such as cell-cycle regulation, genomic imprinting, and cell differentiation [14]. Transcriptome profiling revealed that lncRNAs are expressed at a lower level than protein-coding transcripts and are more developmental stage- and tissue-specific [15]. Studies have confirmed that lncRNAs play a role in adipogenesis. For example, ADINR RNA (adipogenic differentiation induced noncoding RNA, a lncRNA that is transcribed from a position about 450 bp upstream of the CCAAT/enhancer binding protein α [*C/EBPα*] gene) specifically binds PA1 (PAX-Interacting protein 1 [PAXIP1]-associated glutamate rich protein 1 [PAGR1], also known as PA1) and recruits MLL3/4 histone methyltransferase complexes to increase H3K4me3 and decrease H3K27me3 histone modifications at the *C/EBPα* locus, resulting in gene activation and enhanced adipogenesis [16]. Further, the PU.1 antisense lncRNA binds and negatively regulates the level of PU.1 mRNA, suppresses protein expression, and thus improving PU.1-mediated peroxisome proliferator-activated receptor γ (PPARγ) expression [17].

miRNAs are small non-coding RNAs that can function widely by modulating multiple physiological processes such as development, immunity, cell differentiation, apoptosis, metabolism, and signal transduction [18]. These molecules also regulate fat growth, mainly by acting on transcription factors that regulate the signaling pathways involved in adipocyte differentiation or by preventing cell proliferation to promote or inhibit the differentiation of fat cells [19]. Studies have demonstrated that lncRNAs can interact with protein-coding genes through direct competition for miRNA binding [20]. For example, Liu et al [21] found that lncRNA Gm15290 sponges miR-27b to promote PPARγ-mediated adipogenesis *in vitro* and increase fat deposition and body weight in high fat diet-fed mice.

Based on previous high-throughput sequencing studies by our study group, we identified some differentially-expressed lncRNAs in the backfat tissues of Anqing six-end-white (AQ, the obese breed) and Large White (LW, the lean breed) pigs. Among them, we were interested in lncRNA TCONS_ 00010987, because the cis mechanism prediction indicated that its potential target gene was *LEPR*. Considering the physiological and clinical importance of this gene in regulating obesity and the fact that porcine *LEPR* expression has not been previously shown to be under lncRNA control, we conducted a bioinformatic analysis to predict miRNAs that could bind *SSC*-TCONS_00010987-target sequence fragments and the *SSC-LEPR*-3′-UTR. We found that *SSC*-miR-323 could directly bind lncRNA TCONS_00010987 and *LEPR* mRNA with low free energy of binding. Next, we constructed dual luciferase reporter gene vector with an *SSC*-TCONS_00010987 target fragment or an *SSC*-LEPR-3′-UTR, as well as similar vectors with mutations in the respective *SSC*-miR-323-binding sites. The dual luciferase assay system was used to detect relative luciferase activities. Additionally, the backfat tissue expression patterns of lncRNA TCONS_00010987, *SSC*-miR- 323, and *LEPR* in LW and AQ pigs were investigated. This study aimed to provide a theoretical basis for the possible mechanism through which *SSC*-miR-323 and lncRNA TCONS_ 00010987 regulate *LEPR* expression.

## MATERIALS AND METHODS

### Ethics statement

All animal experiments were conducted according to the Regulations and Guidelines for Experimental Animals established by the Ministry of Science and Technology (Beijing, China, revised in 2004) and approved by the Institutional Animal Care and Use Committee of Anhui Agricultural University (Permit number: SYXK 2016-007).

### Animals and tissue sampling

Six purebred castrated male AQ pigs and six purebred castrated male LW pigs (with a body weight of approximately 100 kg) were used in this work. Strictly fast for 24 hours before slaughter, free to drink water. The heart, liver, spleen, lung, kidney, *longissimus dorsi* muscles, and backfat tissues were rapidly harvested from the carcasses and immediately frozen in liquid nitrogen, and all tissue samples were stored at −80°C prior to RNA extraction. The another *longissimus dorsi* muscles were collected to test the content of intramuscular fat.

### Measurements of carcass traits

The backfat thicknesses (mm), intramuscular fat of the *longissimus dorsi* muscles (%), carcass lean meat percentage (%) and loin eye area (cm^2^) of AQ and LW pigs were measured strictly in accordance with the Rules for performance determination of breeding pigs (NY/T 822–2004) issued by the ministry of agriculture of the People’s Republic of China. Intramuscular fat content was determined by measuring the crude fat of muscle using Soxhlet Extraction with petroleum ether.

### Quantitative real-time reverse transcription polymerase chain reaction

Total RNA from the heart, liver, spleen, lung, kidney, longissimus dorsi muscles, and backfat tissues were extracted using the RNAeasy Mini Kit (Qiagen, Hilden, Germany), following the operating manuals. Total RNA was checked for purity and quantity with a Nanodrop 2000 spectrophotometer (Thermo Scientific, Waltham, MA, USA). RNA integrity was measured by denaturing agarose electrophoresis and a Bioanalyzer 2100 system (Agilent Technologies, Santa Clara, CA, USA).

For quantitative analysis of lncRNA TCONS_00010987 and *LEPR* mRNA expression, 1 μg of total RNA from each sample was reverse-transcribed into cDNA using the Prime Script RT Kit (TaKaRa, Tokyo, Japan) at 37°C for 15 min and 85°C for 5 s in a 10-μL reaction mixture, according to the manufacturer’s instructions. The glyceraldehyde-3-phosphate dehydrogenase gene was used as an endogenous control gene.

For the quantitative analysis of *SSC*-miR-323 expression, 1 μg of total RNA from each sample was reverse-transcribed into cDNA using the NCode EXPRESS SYBR GreenER miRNA qPCR Kit (Invitrogen, Carlsbad, CA, USA) at 37°C for 1 h in a 20-μL reaction mixture, according to the manufacturer’s instructions. Porcine U6 small nuclear RNA (U6) was used as an endogenous control gene.

All primers used are listed in Table 1. Quantitative polymerase chain reaction (qPCR) was performed using the CFX96 TouchTM Real-Time PCR Detection System (Bio-Rad, Hercules, CA, USA). Thermal cycling conditions consisted of 95°C for 30 s, followed by 40 cycles at 95°C for 5 s and 60°C for 30 s. Relative lncRNA TCONS_00010987, *LEPR*, and *SSC*-miR-323 expression levels were calculated from Ct values using the 2^−ΔΔCT^ method. All tests were carried out in triplicate.

### Bioinformatics analysis

The porcine LncRNA TCONS_00010987 sequence was obtained from our previous high-throughput sequencing results. The *cis* prediction of target genes was based on the lncRNAs regulating their neighboring or nearby genes. The 10-kb upstream or downstream region of lncRNA TCONS_00010987 was searched for its target gene. According to *cis* prediction, *LEPR* was selected as a potential mRNA target of lncRNA TCONS_00010987 for follow-up studies.

The *SSC*-miR-323 sequence was obtained from the miRDB website (http://www.mirdb.org/mirdb/policy.html) and miRNA–mRNA interactions were obtained from three reliable online miRNA-target databases as follows: BiBiServ (https://bibiserv.cebitec.uni-bielefeld.de/rnahybrid/), miRDB (http://www.mirdb.org/mirdb/policy.html), and RNA22 v2 (https://cm.jefferson.edu/rna22/Interactive/). miRNA-lncRNA interactions were predicted using BiBiServ. Based on the predictive criteria including having good complementarity with targeted sequences, being bound to targeted sequences with low free energy of binding, and being conserved among species, *SSC*-miR-323 was selected as a candidate miRNA that interacts with TCONS_00010987 and *LEPR* for follow-up studies.

### Plasmid construction

Based on the porcine lncRNA TCONS_00010987-target sequence, a partial segment containing the *SSC*-miR-323-bound sequence and a mutated segment of the lncRNA TCONS_ 00010987-target sequence in which the *SSC*-miR-323-bound sequence TAATGTG was converted to ATTACAC were constructed by biosynthesis (Supplementary Figure S1). The expected length of both amplicons was 950 bp. The biosynthesized fragments were inserted between the *XhoI* and *NotI* sites of the pmiR-RB-REPORT^TM^ firefly luciferase reporter vector (RiboBio, Guangzhou, China). Recombinants harboring the desired biosynthesis products were confirmed by DNA sequencing. The recombinant plasmids were termed “WT” (pmiR-RB-REPORT^TM^-*SSC*-TCONS_00010987-wild-type target sequence fragments) and “MT” (pmiR-RB–REPORT^TM^-*SSC*-TCONS_00010987-mutant target sequence fragments).

Based on the porcine *LEPR* mRNA 3′-UTR sequence (NM_ 001024587.1), a partial segment containing the *SSC*-miR-323-bound sequence and a mutated segment in which the *SSC*-miR-323-bound sequence AATGTG was converted to TTACAC were constructed by biosynthesis (Supplementary Figure S2). The expected length of both amplicons was 766 bp. The biosynthesis products were then subcloned into the pmiR-RB-REPORT^TM^ firefly luciferase reporter vector (RiboBio, China) between *XhoI* and *NotI* sites. Recombinants harboring the desired biosynthesis products were confirmed by DNA sequencing. The recombinant plasmids were termed “WT” (pmiR-RB-REPORT^TM^-*SSC*-*LEPR*-wild-3′-UTR) and “MT” (pmiR-RB-REPROT^TM^-*SSC*-*LEPR*-mutant-3′UTR).

*SSC*-miR-323 mimics and the negative control were purchased from RIBOBIO (RiboBio, China).

### Plasmid transfection and luciferase assays

For luciferase assays to test the interaction between *SSC*-TCONS_00010987 and *SSC*-miR-323, HEK293T cells were transiently co-transfected with 100 ng of pmiR-RB-REPORT^TM^-*SSC*-TCONS_00010987-WT or pmiR-RB-REPORT^TM^-*SSC*-TCONS_00010987-MT and 100 nM of SSC-miR-323 mimics or its negative control using Lipofectamine 3000 (Invitrogen, USA) in 96-well plates.

For luciferase assays to test the interaction between *SSC*-*LEPR* and *SSC*-miR-323, HEK293T cells were transiently co-transfected with 100 ng of pmiR-RB-REPORT^TM^-*SSC*-LEPR-WT or pmiR-RB-REPORT^TM^-*SSC*-LEPR-MT and 100 nM *SSC*-miR-323 mimics or its negative control using Lipofectamine 3000 (Invitrogen, USA) in 96-well plates.

Forty-eight hours after transfection, the luciferase activities were determined using the Dual-luciferase Reporter Assay System according to the manufacturer’s instructions (Promega, Madison, WI, USA). All reactions were performed in triplicate.

### Protein extraction and western blotting

Total proteins were extracted from backfat using radio immunoprecipitation assay lysis buffer (Beyotime, Shanghai, China) and protein content was measured using the bicinchoninic acid protein assay kit (Beyotime, China). Total protein (5 to 20 μL) was loaded onto a 10% sodium dodecyl sulfate-polyacrylamide gel electrophoresis gel, separated by electrophoresis, and transferred onto a polyvinylidene difluoride membrane. Blots were blocked with 5% skim milk overnight at 4°C and blotted with specific primary antibodies for LEPR (1:300, bs-20498R, Bioss, Beijing, China) and β-actin (1:2,000, TA-09, ZSGB-BIO, Beijing, China). The bound primary antibodies were determined with a horseradish peroxidase conjugated secondary antibody goat anti-rabbit immunoglobulin G (IgG) (1:2,000, ZB2301, Zsbio, Beijing, China) and a horseradish peroxidase conjugated secondary antibody goat anti-mouse IgG (1:2,000, ZB2305, Zsbio, China), respectively. The data were analyzed using Image J software (GEL system, Beijing kcrx bio-company, Beijing, China). The relative expression of LEPR was calculated as the ration of optical density of LEPR to that of β-actin.

### Statistical analysis

The values are expressed as the mean±standard deviation. A Student’s t-test was used to evaluate the statistical differences between two groups and p<0.05 was considered significant, whereas p<0.01 was considered highly significant.

## RESULTS

### Carcass traits of Anqing Six-end-white and Large White pigs

As shown in Table 2, the average backfat thicknesses of AQ and LW pigs were 45.27±3.25 and 20.07±1.19 mm, respectively. The loin eye area of LW pigs is 107.80% higher than that of AQ pigs (p<0.01), and the lean meat percentage is 55.11% higher than that of AQ pigs (p<0.01). The intramuscular fat of AQ pigs is 177.93% higher than that of LW pigs (p<0.01). Based on the extreme differences in their fat phenotypes, these two breeds comprised good models for our study.

### LncRNA TCONS_00010987 interacts with *SSC*-miR-323

**Figure 1:**
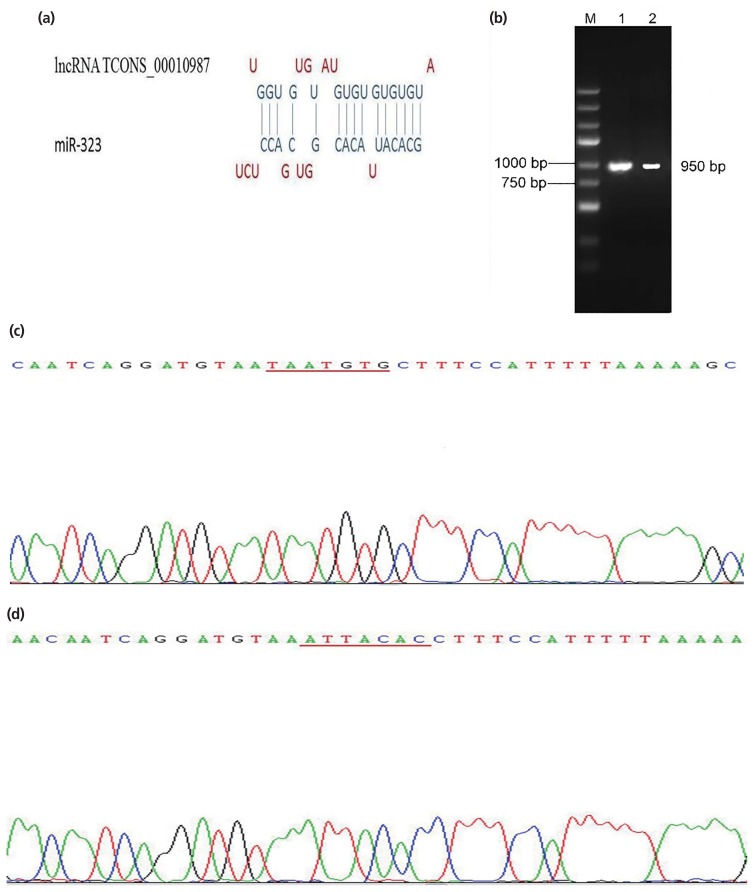
LncRNA TCONS_00010987 is a putative target of *SSC*-miR-323. (a) Representation the lncRNA TCONS_00010987- and miR-323-binding sites by BIBISERV. (b) Identification of PCR fragments by agarose gel electrophoresis. M, DNA marker; lane 1, PCR fragment of the lncRNA TCONS_00010987 target sequence; lane 2, PCR fragment of the lncRNA TCONS_00010987 mutant target sequence. (c) Sequencing results of the dual luciferase reporter vector containing the lncRNA TCONS_00010987 target sequence. (d) Sequencing results of the dual luciferase reporter vector containing the lncRNA TCONS_00010987 mutant target sequence. PCR, polymerase chain reaction.

Based on bioinformatics analyses, we identified one putative binding site between lncRNA TCONS_00010987 and *SSC*-miR-323 (Figure 1a). Gel electrophoresis results showed that the biosynthesis products containing the corresponding target sequence fragments were specific and of the expected size (Figure 1b). The sequencing results of vector containing the *SSC*-TCONS_00010987-wild-type target sequence fragments are shown in Figure 1c; the fragment had 100% sequence identity with the corresponding region of the porcine TCONS_ 00010987 gene obtained by our previous high-throughput sequencing results. Sequencing results of the vector harboring the *SSC*-TCONS_00010987-mutant target sequence fragments are shown in Figure 1d; as shown, the TAATGTG sequence was successfully mutated to ATTACAC, without changes to other bases. These results confirmed that TCONS_00010987-wild-type target sequence fragments and TCONS_00010987-mutant target sequence fragments were successfully cloned into the dual luciferase reporter vectors.

**Figure 2:**
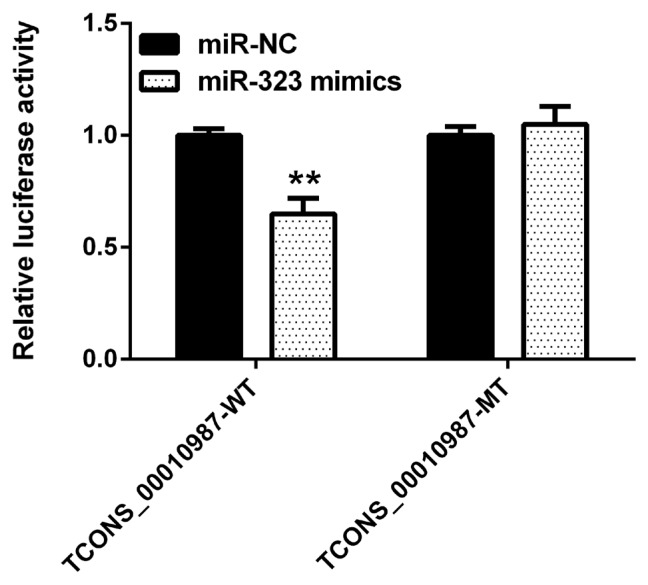
Luciferase activity in HEK293T cells transfected with reporter vectors containing lncRNA TCONS_00010987 target sequence fragment variants and SSC-miR-323 mimics. WT, wild-type TCONS_00010987 target sequence; MT, TCONS_00010987 target sequence with a mutation in the SSC-miR-323-binding site. Data are presented as the mean±standard deviation of n = 3 experiments. ** p<0.01.

As shown in Figure 2, compared to expression with the negative control, *SSC*-miR-323 mimics significantly inhibited luciferase activity from the wild-type *SSC*-TCONS_00010987 target sequence fragments (p<0.01); however, *SSC*-miR-323 mimics had no effect on luciferase activity when the *SSC*-miR-323-binding site was mutated. These results indicated that miR-323 targets TCONS_00010987 via the *SSC*-miR-323- binding site located in the TCONS_00010987-target sequence fragments and inhibits its transcriptional activity.

### Leptin receptor is directly targeted by *SSC*-miR-323

**Figure 3:**
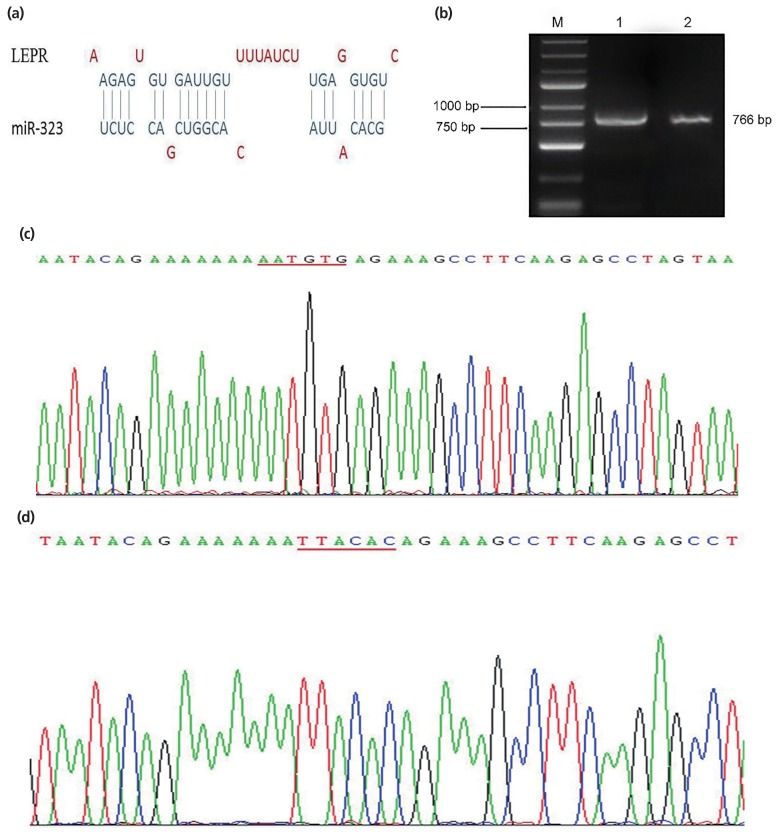
*LEPR* is a putative target of *SSC*-miR-323. (a) Diagram of the putative binding sites between *LEPR* and *SSC*-miR-323 based on bioinformatics prediction. (b) Identification of PCR fragments by agarose gel electrophoresis. M, DNA marker; lane 1, PCR fragment of *LEPR* mRNA 3′-UTR sequence; lane 2, PCR fragment of *LEPR* mRNA 3′-UTR mutant sequence. (c) Sequencing results of the dual luciferase reporter vector containing the *LEPR* 3′-UTR. (d) Sequencing results of the dual luciferase reporter vector containing the *LEPR* 3′-UTR with a target sequence mutation. LEPR, leptin receptor; PCR, polymerase chain reaction.

One putative binding site between *LEPR* and *SSC*-miR-323 was identified using bioinformatic analysis (Figure 3a). Gel electrophoresis results showed that the biosynthesis products containing the corresponding 3′-UTR were specific and of the expected size (Figure 3b). Sequencing results of the *SSC*-*LEPR*-wild-type-3′-UTR vector are shown in Figure 3c, whereas those of the *SSC*-*LEPR*-mutant-3′-UTR vector are shown in Figure 3d; here, the AATGTG sequence was successfully mutated to TTACAC, without changes to other bases. These results confirmed that the *LEPR*-wild-type-3′-UTR and *LEPR*-mutant-3′-UTR were successfully cloned into the dual luciferase reporter vectors.

**Figure 4:**
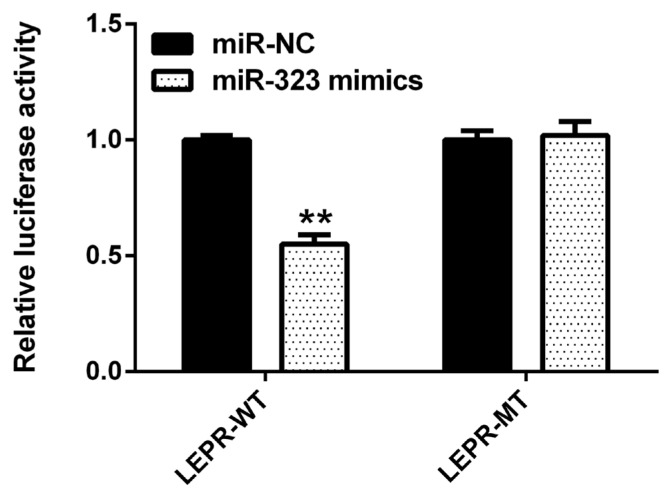
Luciferase activity in HEK293T cells transfected with reporter vectors containing *LEPR* mRNA 3′-UTR variants and *SSC*-miR-323 mimics. WT, wild-type *LEPR* mRNA 3′-UTR; MT, *LEPR* mRNA 3′-UTR with a target sequence mutation in the *SSC*-miR-323-binding site. LEPR, leptin receptor. Data are presented as the mean±standard deviation of n = 3 experiments. ** p<0.01.

As shown in Figure 4, compared to that with the negative control, *SSC*-miR-323 mimics significantly inhibited wild-type *SSC*-LEPR 3′-UTR activity (p<0.01). However, these mimics had no effect on luciferase activity from the *SSC*-*LEPR* mRNA 3′-UTR reporter containing the mutated *SSC*-miR-323-binding site. These results indicated that *SSC*-miR-323 targets *LEPR* via the miR-323-binding site located in the 3′-UTR of the *LEPR* mRNA to inhibit its transcriptional activity.

### Backfat expression patterns of TCONS_00010987, miR-323, and *LEPR* in Anqing Six-end-white and Large White pigs

**Figure 5:**
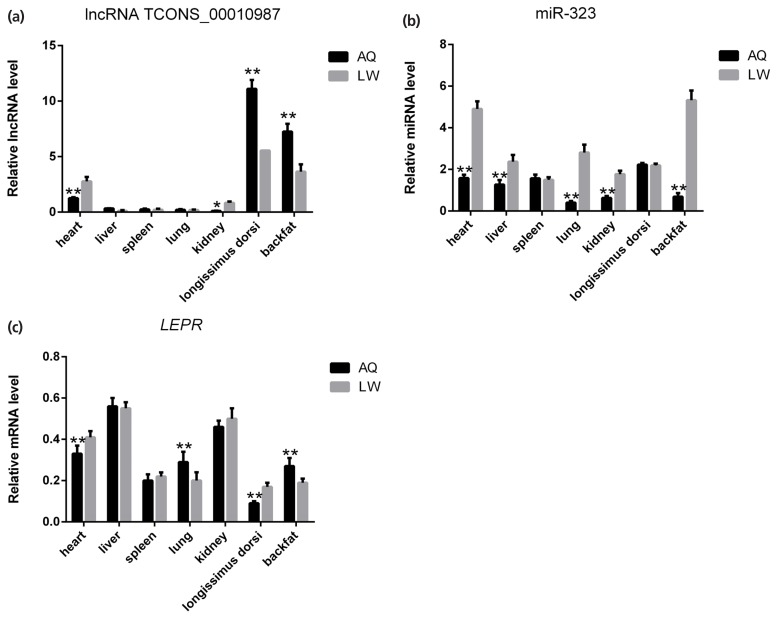
Differential expression of lncRNA TCONS_00010987, miR-323, and target genes in tissues of Anqing Six-end-white and Large White pigs. (a) lncRNA TCONS_00010987; (b) miR-323; (c) *LEPR* mRNA. LEPR, leptin receptor. Data are presented as the mean±standard deviation (n = 6). * p<0.05, ** p<0.01.

**Figure 6:**
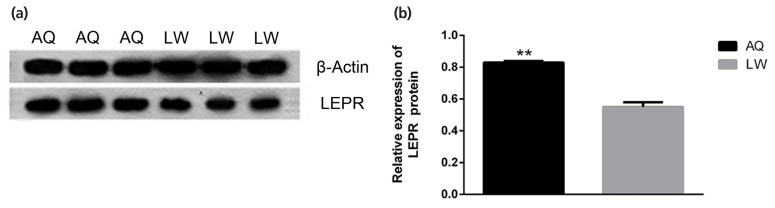
Differential expression of LEPR protein in the backfat of Anqing Six-end-white (AQ) and Large White (LW) pigs. (a) Western blot images. (b) Gray values of western blot results. LEPR, leptin receptor. Data are presented as the mean±standard deviation (n = 3). ** p<0.01.

As shown in Figure 5, qPCR analysis revealed that lncRNA TCONS_00010987, *LEPR*, and *SSC*-miR-323 were all highly expressed in porcine backfat tissues. Furthermore, expression levels of lncRNA TCONS_00010987 and *LEPR* in AQ pigs were significantly higher than those in LW pigs (p<0.01), whereas levels of *SSC*-miR-323 in AQ pigs were significantly lower than those in LW pigs (p<0.01). As shown in Figure 6, western blotting results showed that LEPR protein levels in the backfat tissues of AQ pigs were also markedly higher than those in LW pigs (p<0.01). As indicated, the backfat expression levels of lncRNA TCONS_00010987, *LEPR* mRNA, and LEPR protein in obese pigs were significantly higher than those in the lean breed, whereas levels of *SSC*-miR-323 showed the opposite expression trends.

## DISCUSSION

In this study, we used LW pigs and AQ pigs to comprise lean-fat models based on their significant phenotype differences in carcass fat deposition traits (as shown in Table 2). Lean pig breeds, such as Large White (LW), have been intensively selected over the past decades for improved growth rate and muscularity [22]. AQ pigs, one Chinese fat-type pig breed, have high good meat quality and slow growth rate [23]. Whereas the Chinese domestic breed has the universal characteristics of higher fat deposition capacity compared to that with Western commercial pig breeds [24]. Accordingly, the two pig breeds can be taken as ideal animal models to comparatively study the mechanisms controlling adipogenesis.

Leptin receptors interact with leptin to play a wide range of roles in animals, such as controlling the body mass balance, regulating body fat content and regulating feed intake [25]. Adipocytes synthesize and secrete leptin, which in turn acts on the hypothalamus to maintain the stability of body fat [8]. At high levels, leptin inhibits food intake through the melanocortin receptor system, and at low levels, it promotes food intake through neuropeptide Y. Mutations in the *LEPR* gene result in leptin insensitivity, hyperphagia, morbid obesity, and metabolic and endocrine abnormalities [26]. In pigs, LEPR mRNA was expressed in adipose tissue, brain, muscle, fat, liver, hypothalamus, pituitary gland and reproductive tissues (endometrium and ovary) [27]. Studies have confirmed that the expression of LEPR in the muscle and fat tissues in high-fat pigs is significantly higher than that in low-fat pigs [28,29]. Based on our results, we confirmed this; specifically, LEPR protein and mRNA expression levels were both significantly higher in the backfat of obese breed pigs (AQ) than in that of lean breed pigs (LW). It was speculated that the increase of LEPR expression was caused by increased fat deposition, which promoted the decomposition of fat and controlled the body mass balance in pigs.

Su et al [30] showed that *SSC*-miR-323 is significantly increased in the myocardial tissue of pigs with coronary microembolization. Further, Skovgaard et al [31] found that miR-323 might be involved in controlling acute influenza infection in pigs. However, to date, there had been virtually no research on miR-323 in pig fat. Based on our results, *SSC*-miR-323 was expressed in the backfat tissues of both AQ and LW pigs; however, the expression patterns in obese and lean pigs differed, indicating that *SSC*-miR-323 might be associated with fat metabolism in pigs.

LncRNA TCONS_00010987 is a novel lncRNA, for which the sequence was obtained based on our previous high-throughput sequencing; moreover, the coding potential calculator analysis suggested coding potential to have a score <0 [32], demonstrating that it is a non-coding RNA. The results of this experiment showed that lncRNA TCONS_00010987 expression levels in the backfat differ greatly between the AQ and LW pigs. Specifically, we demonstrated that whereas lncRNA TCONS_00010987 is indeed expressed in this tissue, expression levels differ depending on whether the animal is obese or lean.

Increasing studies have reported that many lncRNAs function as competing endogenous RNAs (ceRNAs) by serving as sponges that bind and sequester miRNAs. For example, Lv et al [32] revealed that lncRNA Unigene56159 promotes epithelial–mesenchymal transition by acting as a ceRNA for miR-140-5p in hepatocellular carcinoma cells. Further, Zhao et al [33] provided evidence that lncRNA-plasmacytoma variant translocation 1 functions as an endogenous ‘sponge’ by competing for miR-448 binding to regulate expression of the target *SERBP1*, thereby promoting the proliferation and migration of pancreatic cancer cells. Finally, Shang et al. found that lncRNA TCONS_00041960 enhances osteogenesis and inhibits adipogenesis in rat bone marrow mesenchymal stem cells by targeting miR-204-5p and miR-125a-3p [34].

In the present study, bioinformatics analyses predicted a link among porcine lncRNA TCONS_00010987, *SSC*-miR-323, and *LEPR*. Luciferase-reporter assays confirmed that lncRNA TCONS_00010987 might act as a sponge for *SSC*-miR-323 to regulate *LEPR* expression. In addition, qPCR and western blot analysis indicated that the backfat expression patterns of lncRNA TCONS_00010987, *LEPR* mRNA, and LEPR protein in obese and lean pigs were opposite to those of *SSC*-miR-323.

In conclusion, luciferase activity and expression pattern assays indicated that lncRNA TCONS_00010987 might act as a sponge for *SSC*-miR-323 to regulate *LEPR* expression. These results, suggesting that *SSC*-miR-323 can bind *LEPR* and TCONS_00010987, indicate that further study is required to fully elucidate the role of *SSC*-miR-323, TCONS_00010987, and *LEPR* in the epigenetic mechanisms that affect fat metablism in pigs. These results have implications in understanding the detailed mechanisms controlling adipogenesis, energy homeostasis, and obesity.

## Supplementary Data



## Figures and Tables

**Table 1:** Specific primer sequences for real-time quantitative polymerase chain reaction

Primer name	Primer sequences(5′–3′)
TCONS_00010987	F: GGTTTTCACACAGAACAATCAGG
	R: GACCAGCCAAAGGGTATCAA
LEPR	F: ATTCCTGATTCCGTGGTGAA
	R: CGGGAGACTGGTGGATTT
miR-323	F: CTGCACATTACACGGTCGACCT
GAPDH	F: GGAGAACGGGAAGCTTGTCA
	R: GGTTCACGCCCATCACAAAC
U6	F: CTCGCTTCGGCAGCACA
	R: AACGCTTCACGAATTTGCGT

LEPR, leptin receptor; GAPDH, glyceraldehyde-3-phosphate dehydrogenase.

**Table 2:** Carcass traits of Anqing Six-end-white and Large White pigs

Traits	Anqing Six-end-white (n = 6)	Large White (n = 6)
Backfat thicknesses (mm)	45.27±3.25^A^	20.07±1.19^B^
Intramuscular fat (%)	5.92±0.41^A^	2.13±0.30^B^
Lean meat percentage (%)	43.68±2.49^B^	67.75±3.18^A^
Loin eye area (cm^2^)	28.06±1.24^B^	58.31±2.15^A^

Data are presented as the mean±standard deviation (n = 6).

A,B Different uppercase letters in the same row represent significant differences between samples (p<0.01).
