# Supplementary material for: Relationship among porcine lncRNA TCONS_00010987, miR-323, and leptin receptor based on dual luciferase reporter gene assays and expression patterns
Source: Asian-Australas J Anim Sci. 2019 Jul 1;33(2):219–29. doi: 10.5713/ajas.19.0065 (PMC6946967; doi:10.5713/ajas.19.0065)
Supplement: Supplementary file 1 [file ajas-19-0065-suppl.pdf]

(a)

GGCATTTCCGGCCAGGGAGGACGCTCCAGATGAATGGGTAAGTACATCAAGAGCTTCGTGGAGCGC
GTGCTGAAGAACGAGCAGTAATTCTAGGCGATCGCTCGAGAGGGAGACTGACTCTACTCGTGGGCC
AATAATTTCTGTGAAGTTTATGGGAACGTATACGTTTGTACTAGATGATACGAACATATCTGTTGGTAC
TAGATGATAGCTAAGGGTTTGCGCCAAGTAGAGCTTCTCAGGAGCCTCTATAATGGTGTTCTAATAGG
CTTTACGAATGTGAACTCATGTTGAAGAACAGCTGTTACCAAATAGCTGTTTCATGAAGGCGGGGGA
CAGCTTAGTTTTTCTCAGAATTATATTTTCTCATTTTACTTTAACTAGCAGCTGTTATTTGGATGTCTAC
ACCAATGAGATAATCAAGCAATTAGATTTCTGTATGCACCAGCAAACCAGTCAGTGTGTAGGAGATG
GAAGAGGAAATAAATCACACACATGTATGGTGTGTATGTGTGTGTGTAGGCATAAACAGAGAAAAAA
CGTACTGGCACATACATAATAAGCACCTGCTAATTGTAGGTGTCATAATACTAAGATGTTATTTCTTAG
GGTTATGACGTATTTGTCATATTGGACCTACCCAGGAGGTAGCTGAATATTTCTGCAGTGATCTGGGT
GTCTTGCAGGGAGCACTGCTGGTCACAGGTCTGATATGAGGGAAGAAAAGGTTTTCACACAGAACA
ATCAGGATGTAATAATGTGCTTTCCATTTTTAAAAAGCAGTCATCTGGGCAAGTGGCTTTTTGGTCTTT
TGACCAACACTGTTCTCAACTTGCAAATTAATTCTTAGGCAGTTCTTACACAGCGGCCGCTGGCCGC
AATAAAATATCTTTATTTTCATACATCTGTGTGTTGTTTATAAAGC

(b)

ACTGGATTGGTCATGCAGAGCTCCAGATGAATGGGTAAGTACATCAAGAGCTTCGTGGAGCGCGTGC
TGAAGAACGAGCAGTAATTCTAGGCGATCGCTCGAGAGGGAGACTGACTCTACTCGTGGGCCAATA
ATTTCTGTGAAGTTTATGGGAACGTATACGTTTGTACTAGATGATACGAACATATCTGTTGGTACTAGA
TGATAGCTAAGGGTTTGCGCCAAGTAGAGCTTCTCAGGAGCCTCTATAATGGTGTTCTAATAGGCTTT
ACGAATGTGAACTCATGTTGAAGAACAGCTGTTACCAAATAGCTGTTTCATGAAGGCGGGGGACAG

CTTAGTTTTTCTCAGAATTATATTTTCTCATTTTACTTTAACTAGCAGCTGTTATTTGGATGTCTACACC
AATGAGATAATCAAGCAATTAGATTTCTGTATGCACCAGCAAACCAGTCAGTGTGTAGGAGATGGAA
GAGGAAATAAATCACACACATGTATGGTGTGTATGTGTGTGTGTAGGCATAAACAGAGAAAAAACGT
ACTGGCACATACATAATAAGCACCTGCTAATTGTAGGTGTCATAATACTAAGATGTTATTTCTTAGGGT
TATGACGTATTTGTCATATTGGACCTACCCAGGAGGTAGCTGAATATTTCTGCAGTGATCTGGGTGTCT
TGCAGGGAGCACTGCTGGTCACAGGTCTGATATGAGGGAAGAAAAGGTTTTCACACAGAACAATCA
GGATGTAAATTACACCTTTCCATTTTTAAAAAGCAGTCATCTGGGCAAGTGGCTTTTTGGTCTTTTGAC
CAACACTGTTCTCAACTTGCAAATTAATTCTTAGGCAGTTCTTACACAGCGGCCGCTGGCCGCAATA
AAATATCTTTATTTTCATTACATCTGTGTGTTGGGTTTTTTTGTGTGAGGATCTAAATGAGTCTTTCGG
ACCCTCGCGGGGGGCCGCTTAAGCGGTGGTTAGGGTTTGT

Figure S1.

(a)

GGAGAGCTCCAGATGAATGGGTAAGTACATCAAGAGCTTCGTGGAGCGCGTGCTGAAGAACGAGC
AGTAATTCTAGGCGATCGCTCGAGGTTCATTCCAGAAACATCTCAGATTTATGATGGGATGAGTCAT
ATTAAGGGTAATATGTTCTACATGGTGTTCCATAGCAGAGAGAAAAAAATTGAGTCAAATTTGAAA
ATGACTTCAAAAGTTAAAGAGATCTGTTTGTCCACACTCAGTAATACAGAAAAAAAAATGTGAGAA
AGCCTTCAAGAGCCTAGTAATGTAGACCTACTCTTCTAATGATTCTCTTAACCGGCTACAGTGGGAA
GTTCTCGAATGCCTTGTGTCTAGCTAGAAACAAGCCCAACAATACTAGCGTTTTGAGCATTAATCTC
ATGTAGAAAGAGCTAATCCATCTGAATTACACATACATCTGAAAGAAGACTTCAGACTAACACTTG
TGAAATGTAATGTCTTCAAGAGTGTGATTGTTTTATCTTGAGGTGTCTTTGTTTTACACTAATTTACA
CATACACATATGCACACTTGTATCTAATAGGCATCCTGTACATTGTTAAATATATGATGTACTTGTTT
TTGTGCTGCGGCCGCTGGCCGCAATAAAATATCTTTATTTTCATTACATCTGTGTG

(b)

GGAGAGCTCCAGATGAATGGGTAAGTACATCAAGAGCTTCGTGGAGCGCGTGCTGAAGAACGAGC
AGTAATTCTAGGCGATCGCTCGAGGTTCATTCCAGAAACATCTCAGATTTATGATGGGATGAGTCAT
ATTAAGGGTAATATGTTCTACATGGTGTTCCATAGCAGAGAGAAAAAAATTGAGTCAAATTTGAAA
ATGACTTCAAAAGTTAAAGAGATCTGTTTGTCCACACTCAGTAATACAGAAAAAAATTACACAGAA
AGCCTTCAAGAGCCTAGTAATGTAGACCTACTCTTCTAATGATTCTCTTAACCGGCTACAGTGGGAA
GTTCTCGAATGCCTTGTGTCTAGCTAGAAACAAGCCCAACAATACTAGCGTTTTGAGCATTAATCTC
ATGTAGAAAGAGCTAATCCATCTGAATTACACATACATCTGAAAGAAGACTTCAGACTAACACTTG
TGAAATGTAATGTCTTCAAGAGTGTGATTGTTTTATCTTGAGGTGTCTTTGTTTTACACTAATTTACA
CATACACATATGCACACTTGTATCTAATAGGCATCCTGTACATTGTTAAATATATGATGTACTTGTTT

TTGTGCTGCGGCCGCTGGCCGCAATAAAATATCTTTATTTTCATTACATCTGTGTGT

Figure S2.

Supplemental Figure legends

Figure S1. LncRNA target sequence fragments as constructed by biosynthesis. (a) *SSC*-TCONS_00010987-WT; (b) *SSC*-TCONS_00010987-MT. The black line indicates an insertion sequence, the carrier sequence is on both sides, and the target sequence is yellow.

Figure S2. *LEPR* 3′-UTR fragments as constructed by biosynthesis. (a) *SSC*-LEPR-WT; (b) *SSC*-LEPR-MT. The black line is labeled as an insertion sequence, the carrier sequence is on both sides, and the target sequence is yellow.
